# Enhanced use of gaze cue in a face-following task after brief trial experience in individuals with autism spectrum disorder

**DOI:** 10.1038/s41598-021-90230-6

**Published:** 2021-05-27

**Authors:** Takao Fukui, Mrinmoy Chakrabarty, Misako Sano, Ari Tanaka, Mayuko Suzuki, Sooyung Kim, Hiromi Agarie, Reiko Fukatsu, Kengo Nishimaki, Yasoichi Nakajima, Makoto Wada

**Affiliations:** 1 Department of Rehabilitation for Brain Functions, Research Institute, National Rehabilitation Center for Persons with Disabilities, 4-1 Namiki, Tokorozawa, Saitama 359-8555 Japan; 2 Information and Support Center for the Persons with Developmental Disabilities, National Rehabilitation Center for Persons with Disabilities, Tokorozawa, Japan; 3 Department of Medical Treatment III (Pediatric and Child Psychiatric Section), Hospital, National Rehabilitation Center for Persons with Disabilities, Tokorozawa, Japan; 4 Present Address: Faculty of Systems Design, Tokyo Metropolitan University, 6-6 Asahigaoka, Hino, Tokyo 191-0065 Japan; 5 Present Address: Department of Social Sciences and Humanities, Indraprastha Institute of Information Technology Delhi, New Delhi, India; 6 Present Address: Occupational Therapy Sciences, Department of Integrated Health Sciences, Graduate School of Medicine, Nagoya University, Nagoya, Japan; 7 Present Address: National Rehabilitation Center for Persons with Disabilities, Tokorozawa, Japan; 8 Present Address: Community Health Care Research Center, Nagano University of Health and Medicine, Nagano, Japan

**Keywords:** Neuroscience, Psychology

## Abstract

Eye movements toward sequentially presented face images with or without gaze cues were recorded to investigate whether those with ASD, in comparison to their typically developing (TD) peers, could prospectively perform the task according to gaze cues. Line-drawn face images were sequentially presented for one second each on a laptop PC display, and the face images shifted from side-to-side and up-and-down. In the gaze cue condition, the gaze of the face image was directed to the position where the next face would be presented. Although the participants with ASD looked less at the eye area of the face image than their TD peers, they could perform comparable smooth gaze shift to the gaze cue of the face image in the gaze cue condition. This appropriate gaze shift in the ASD group was more evident in the second half of trials in than in the first half, as revealed by the mean proportion of fixation time in the eye area to valid gaze data in the early phase (during face image presentation) and the time to first fixation on the eye area. These results suggest that individuals with ASD may benefit from the short-period trial experiment by enhancing the usage of gaze cue.

## Introduction

Autism spectrum disorder (ASD) was first identified by Leo Kanner^1^ and Hans Asperger^2^ at almost the same time, nearly 80 years ago. Although its etiology is not yet fully known, this developmental disorder is characterized by deficits in social interactions, communication, and imagination^3–5^. ASD has traditionally been regarded as a social and cognitive disorder^6–9^, and previous studies have demonstrated that individuals with ASD also show different patterns of gaze motor behavior^10–14^ and joint attention^15,16^ to those of their typically developing (TD) peers.

Individuals with ASD often avoid eye contact when observing a face^12,17^, while their TD peers tend instead to scan eye and mouth areas^18^. Dalton et al.^17^ found that eye fixation time is positively associated with amygdala activation in individuals with ASD (and not their TD peers) (approximate mean age = 15 ± 5 years) during a task in which participants had to explore the eye region of a face. Furthermore, Kylliäinen and Hietanen^19^ demonstrated that school-aged children with ASD (mean age = 8 years 11 months ± 2 years 10 months) exhibited greater galvanic skin responses (i.e., enhanced arousal) to eyes with a straight gaze (in comparison to eyes with an averted gaze), but this was not the case in the control group (see also Joseph et al.^20^). These results imply that individuals with ASD adopt a modulation strategy to reduce this over-arousal (which emerges as amygdala hyperactivation and greater galvanic skin responses) by avoiding eye contact, which is a primary symptom of the ASD.

Joint attention is a key function of social cognition. Butterworth and Jarrett^21^ have defined joint attention as adjusting one’s own attention based on a change in another’s focus of attention or, more simply, looking where someone else is looking (see also Mundy and Newell^22^). Responding to joint attention has also been referred to as “gaze following”. Senju, Tojo, Dairoku, and Hasegawa^23^ investigated whether gaze cue (a social cue) and an arrow (a non-social directional cue) trigger reflexive attention-orienting in children with and without ASD, using almost the same paradigm of Driver et al.^24^. They found that participants with ASD (aged 7.6 to 12.3 years) were slower to respond, but shifted attention to the cued location using both social and non-social cues; in comparison, TD children showed specific quicker responses by gaze cue—not by the arrow (see also studies involving more complex environments^25,26^). These results suggest that individuals with ASD have no general impairment in visual attention and no specific impairment in gaze orienting per se; rather, they have a deficit in social cue perception^27,28^.

Recent studies have noted that the development of motor skills is strongly associated with both cognitive and social development^29,30^. Casartelli, Molteni, and Ronconi^31^ have also argued that the cortical motor system can play a role in complex social cognition^32^. Indeed, gaze behavior includes eye movements, which are also motor functions. According to this motor-social argument, social communication skills should be improved by sensorimotor training. As the first step of the training program development in this study, we investigated how older adolescents and young adults with ASD perform a task in which they are required to use gaze cues that require fixating on the eye area of an image of a face. Specifically, we explored whether a short-period trial experiment that required the use of gaze cues to perform the task efficiently enhanced the appropriate use of gaze cues (evaluated by gaze behavior) in individuals with ASD who have difficulty with eye contact. To this aim, we separated data into those from the first and second half of the trials. The present study focused on the motor aspects of the task rather than the mere perceptual ones; therefore, we used a face-following task with gaze cue in a sequential manner (see “Methods” for details), whereas many previous studies^23,24,33,34^ have employed a “one-shot” cueing paradigm. Furthermore, we investigated the relationship between face-following performance and Autism-Spectrum Quotient (AQ) scores in both TD and ASD groups.

## Results

**Figure 1:**
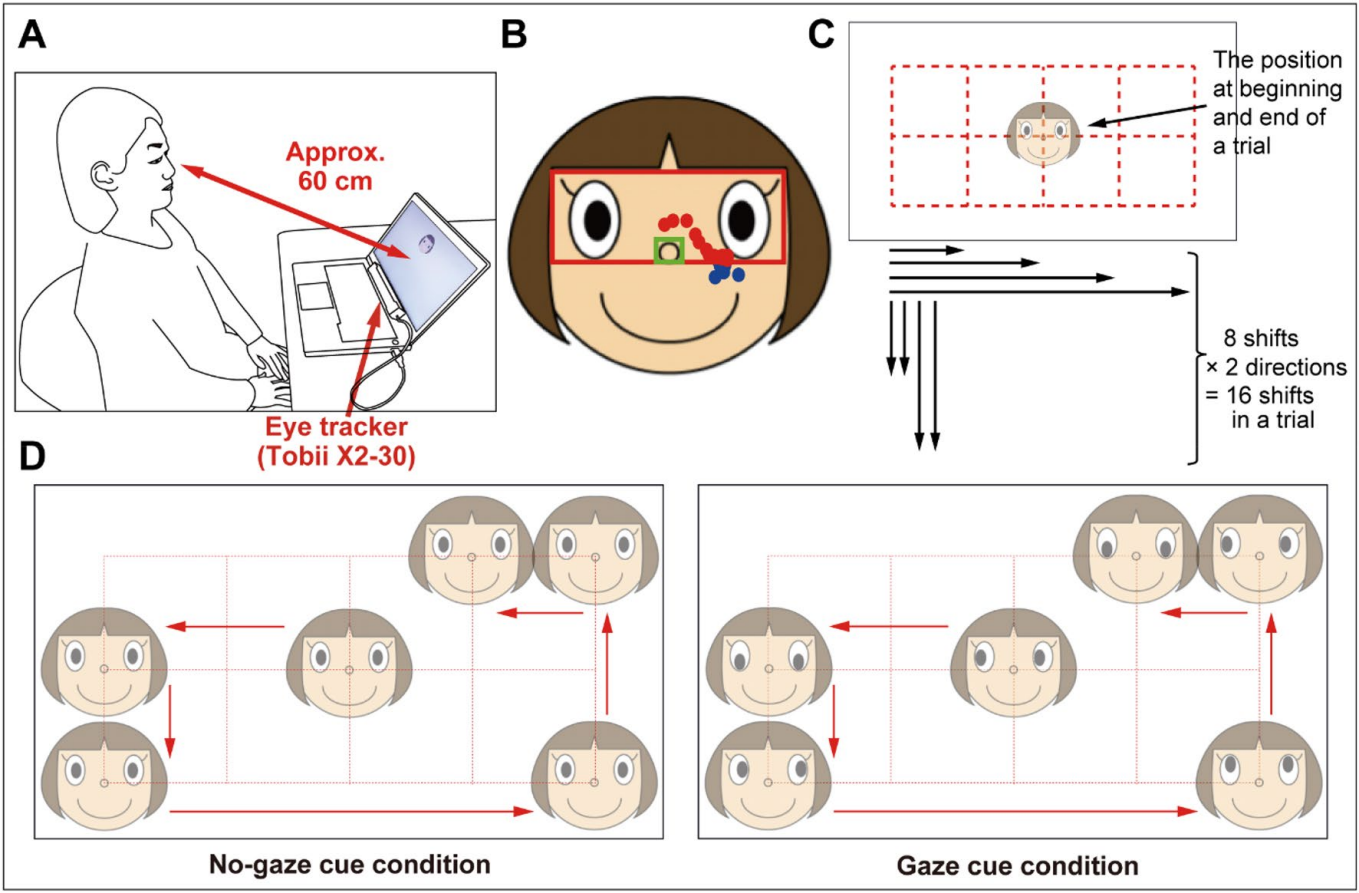
Experimental setup (**A**). Line-drawn smiling face image. Red and green rectangles denote the eye and nose areas, respectively. Red dots denote the data included for the fixation time analysis, and blue dots denote the data excluded from fixation time analysis (**B**). Potential locations for presenting face images. The face image was always presented in the center of the screen at the beginning and end of each trial. Each trial consisted of 16 displacements; these included four distances of both directions for horizontal displacement, and two distances of both directions for vertical displacement (twice for each distance and direction in a single trial) (**C**). Experimental conditions (**D**).

We recorded eye movements toward sequentially presented face images with or without gaze cues (Fig. 1). Face images in each sequence were presented for one second, and fixation time in the eyes area was calculated.

Before calculating the fixation time, two “sanity check” analyses were performed. First, the duration in which fixation could not be detected by the eye-tracker was calculated. In the present study, a chin rest was not used to stabilize the participants’ eye positions because the eye-tracker is considered to be robust to head motion. However, despite performing a thorough calibration in each participant, the participants could perform eye-tracking behavior in a “peculiar” manner (e.g., with head movement and squinting), which disrupted a proper recording. The mean ratio of this duration of no valid gaze data to the one-second presentation time was computed and the results (mean ± SD) were as follows: 24.1 (± 17.9)% for the gaze cue condition and 14.2 (± 9.2)% for the no-gaze cue condition in the ASD group, and 14.1 (± 8.9)% for the gaze cue condition and 7.6 (± 6.2)% for the no-gaze cue condition in the TD group. A two-way analysis of variance (ANOVA) with group (ASD, TD) as a between-participants factor and the gaze-cue condition (gaze and no-gaze cue conditions) as a within-participant factor revealed a significant main effect of gaze condition [*F*(1, 18) = 11.654, *p* = 0.003, partial *η*^2^ = 0.393]. There was no significant main effect of group [*F*(1, 18) = 2.960, *p* = 0.103] and no significant interaction [*F*(1, 18) = 0.458, *p* = 0.507]. This duration was excluded from the following analysis.

Next, the number of sequences for which the fixation point never dropped within the eye area (i.e., the fixation time in the eye area was zero) during face stimulus presentation was counted to examine whether participants adopted a specific task strategy (such as not directly fixating on the eye area by dissociating attentional direction from gaze direction), or simply avoided fixating on the eye area (especially in the ASD group). There were a total of 160 sequences (16 sequences × 10 trials) and the ratio of “zero-fixation to eye area” sequences to total sequences were computed separately for the first and second half of trials (i.e., each of 5 trials (80 sequences)). A three-way ANOVA with group (ASD, TD) as a between-participants factor and the gaze-cue condition (gaze and no-gaze cue conditions) and period (first 5 trials and second 5 trials) as within-participant factors revealed a significant main effect of group [*F*(1, 18) = 18.028, *p* = 0.001, partial *η*^2^ = 0.500; ASD: 31.7 (± 14.4)%; TD: 11.0 (± 5.4)%]. No other significant main effects or interactions were found (*ps* > 0.474). These “zero-fixation to eye area” sequences were not included in the following fixation time analysis.

Concerning the fixation time to the eye area of the face stimuli, data was separated into time bins of 0.2 s and the proportion of fixation time was calculated, as shown below (Supplementary Fig. S1 shows the mean proportion of fixation time in the eye area to valid gaze data during each 0.2 s time window from all 10 trials, in both groups and both gaze-cue conditions).

Proportion of fixation time to eye area = *Fixation time to the eye area (s)/Valid fixation time on screen (s)*

**Figure 2:**
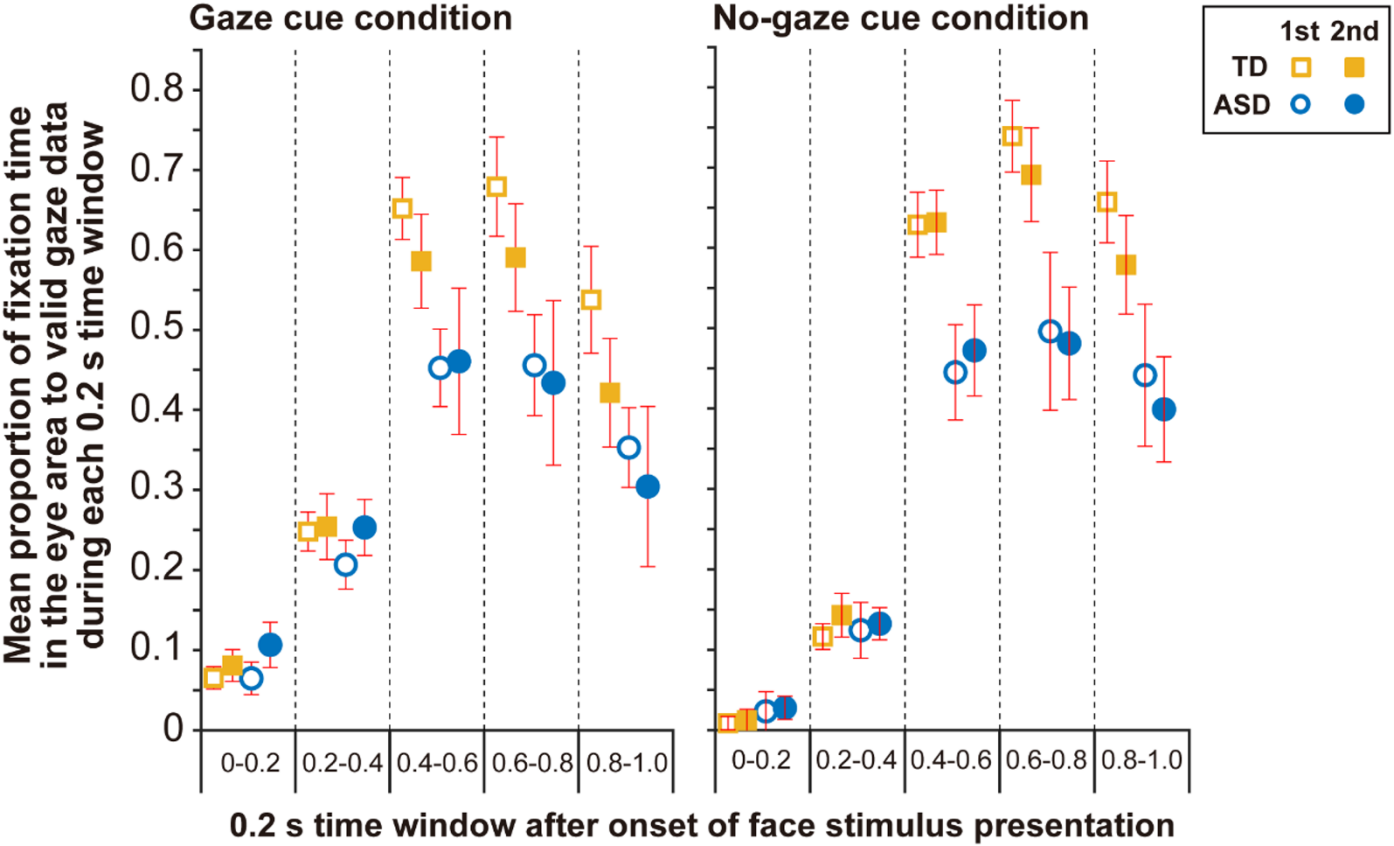
Mean proportion of fixation time in the eye area in each 0.2 s time window, separated into the first 5 trials and second 5 trials. We found a larger proportion of fixation time in the eye area in the second 5 trials than in the first 5 trials during the 0–0.2 s and 0.2–0.4 s time windows after the onset of face image presentation. There was a smaller proportion of fixation time in the eye area in the second 5 trials than in the first 5 trials during the 0.8–1.0 s time window. Error bars indicate 95% within-participant confidence intervals^35,36^.

Given that our present focus was to examine whether and how fixation to the eyes are modulated by a short-period gaze-following task, we divided data obtained during the first and second half of trials. Figure 2 shows the mean proportion of fixation time in the eye area to valid gaze data during each 0.2 s time window in the first half and second half of trials for both groups and gaze-cue conditions. To determine how the gaze-cue and trial experience influence gaze performance of participants with or without ASD in each time window, the mean proportion of fixation time in the eye area in each 0.2 s bin was entered into a three-way ANOVA with the group (ASD, TD) as a between-participants factor and the gaze-cue condition (gaze and no-gaze cue conditions) and period (first 5 trials and second 5 trials) as within-participant factors. We performed ANOVAs for all time-bins (i.e., 0–0.2, 0.2–0.4, 0.4–0.6, 0.6–0.8, and 0.8–1.0 s after stimulus presentation), so α = 0.05/5 = 0.01 was considered to indicate statistical significance using the Bonferroni correction.

Significant main effects of the gaze-cue condition were found in the 0–0.2 s, 0.2–0.4 s, and 0.8–1.0 s time windows (*p*s < 0.0029); specifically, the proportion in the gaze cue condition was significantly larger than that in the no-gaze cue condition during 0–0.2 s and 0.2–0.4 s time windows, while the proportion in the gaze cue condition was significantly smaller than that in the no-gaze cue condition during the 0.8–1.0 s time window. Significant main effects of period were revealed in the 0–0.2 s, 0.2–0.4 s, and 0.8–1.0 s time windows (*p*s < 0.0035); specifically, the proportion in the second 5 trials were significantly larger than that in the first 5 trials during the 0–0.2 s and 0.2–0.4 s time windows, while the proportion in the second 5 trials were significantly smaller than that in the first 5 trials during the 0.8–1.0 s time window. There was a significant main effect of group in the 0.4–0.6 s, 0.6–0.8 s, and 0.8–1.0 s time windows (*p*s < 0.0003), whereby the TD group had significantly larger proportions than the ASD group in these time windows.

**Figure 3:**
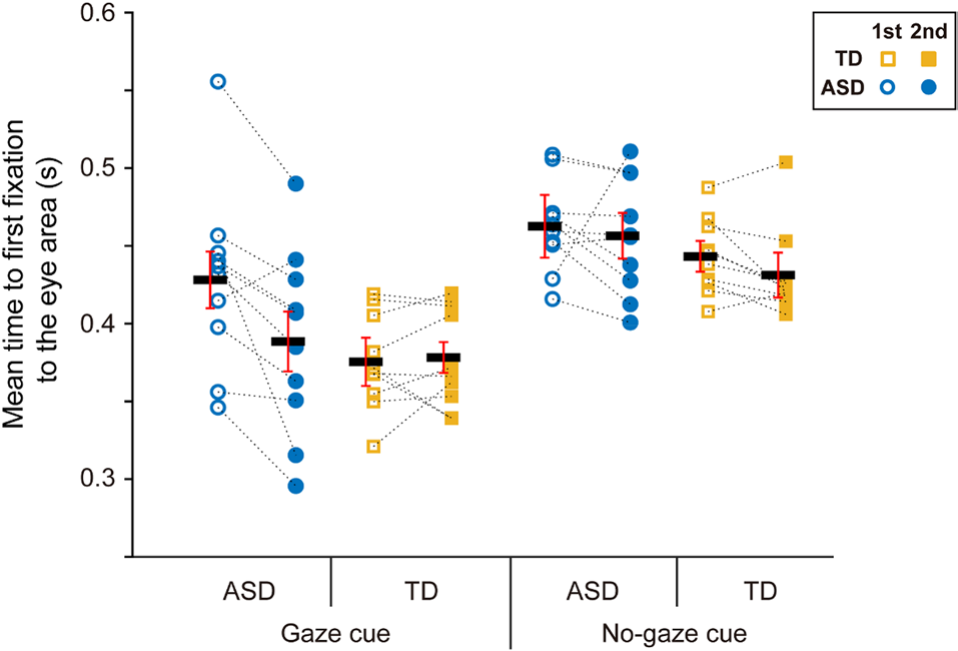
Mean time to first fixation to the eye area of the presented face image. The ASD participants had quicker and comparable (to TD peers) responses to the eye area in the second 5 trials compared with the first 5 trials. Black bars indicate the mean values in each condition. Error bars indicate 95% within-participant confidence intervals.

In addition to the mean proportion of fixation time in the eye area in each 0.2 s bin, we also analyzed the time to first fixation to the eye area of the presented face image (Fig. 3). A three-way ANOVA with group (ASD, TD) as a between-participants factor and the gaze-cue condition (gaze and no-gaze cue conditions) and period (first 5 trials and second 5 trials) as a within-participant factor revealed a significant main effect of gaze-cue condition [*F*(1, 18) = 31.557, *p* < 0.001, partial *η*^2^ = 0.637; gaze cue condition: 0.393 ± 0.046 s; no-gaze cue condition: 0.448 ± 0.029 s] and a significant main effect of period [*F*(1, 18) = 8.485, *p* = 0.009, partial *η*^2^ = 0.320; first 5 trials: 0.427 ± 0.033 s; second 5 trials: 0.414 ± 0.034 s]. A main effect of group almost reached significance [*F*(1, 18) = 4.315, *p* = 0.052, partial *η*^2^ = 0.193]. Furthermore, there was a significant second-order interaction between group, gaze-cue condition, and period (group × gaze-cue condition × period interaction) [*F*(1, 18) = 6.188, *p* = 0.023, partial *η*^2^ = 0.256]. A post-hoc comparison revealed that the time to first fixation to the eye area for the first 5 trials of the gaze cue condition was significantly longer in the ASD group (0.428 ± 0.055 s) than in the TD group (0.375 ± 0.029 s), while no significant difference between the ASD and TD groups was noted in the second 5 trials in the gaze cue condition (ASD: 0.389 ± 0.056 s; TD: 0.378 ± 0.030 s).

To examine whether the total gaze duration to the eye area during face image presentation is associated with the time to first fixation, and whether this association was different between the ASD and TD groups, Fisher-transformed correlations (*r*′) between the total gaze duration and the time to first fixation for each condition in each participant were calculated. The mean *r*′ for all conditions was − 0.437 (± 0.073) in the ASD group and − 0.514 (± 0.100) in the TD group. Two-tailed t-tests revealed that each *r’* was significantly different from 0 (ASD: *t*(9) = − 19.026, *p* < 0.001; TD: *t*(9) = − 16.182, *p* < 0.001). Next, the *r’* for each condition in each participant was entered into a three-way ANOVA with group (ASD, TD) as a between-participants factor, and the gaze-cue condition (gaze and no-gaze cue conditions) and period (first 5 trials and second 5 trials) as within-participant factors. A main effect of group almost reached significance [*F*(1, 18) = 3.779, *p* = 0.068, partial *η*^2^ = 0.174], and no other significant main effects or interactions were found (*p*s > 0.400).

To clarify the relationship between gaze behavior and autistic traits (i.e., total AQ score), Pearson’s correlation coefficients between the total AQ score and the index of gaze behavior were computed in each time-bin (by pooling data of both the ASD and TD groups). This analysis revealed a significant correlation in 0–0.2 s time window only (*r* = 0.572, *p* = 0.008); no significant correlations were found in the other time-bins (*p*s > 0.118) (Supplementary Fig. S2). In the 0–0.2 s time-window, there seemed to be one outlier point [36, 4.22]; after removing this point, the correlation was still relatively strong (*r* = 0.458) but no longer significant (*p* = 0.049; in this case, α was 0.01; see Data Processing and Analyses for details).

## Discussion

This eye-tracking study explored how older adolescents and adults with ASD perform a face-following task based on a gaze cue (with the gaze directed to the position at which the next face would be presented) or no gaze cue (gaze directed to participants), compared with their TD peers. We also examined whether accumulating trial experience influences gaze behavior in a relatively short time (i.e., 16 sequences × 10 trials; less than 15 min) by separately analyzing data for the first and second 5 trials, and whether the face-following performance (according to the gaze cue and trial experience) is associated with the total AQ scores among TD individuals and those with ASD.

As reported by previous studies^10,12^, the ASD group looked less at the eye area in the face images. Specifically, the fixation time in the eye area in the ASD group diverged from that in the TD group from around the second half of the one-second face image presentation (i.e., the 0.4–0.6 s time-window) and the mean *accumulated* fixation time in the eye area (not shown in Fig. 2 and Supplementary Fig. S1) in the ASD group was around half of their TD peers. The ratio of “zero-fixation to the eye area” sequences to the total sequences also confirmed this “less frequent looking at the eyes” gaze property in the ASD group.

However, fixation enhancement in the ASD group by the gaze cue in the early phase during face image presentation was comparable to the TD group, as revealed by the main effect of gaze-cue; specifically, there was a significantly larger proportion of fixation time in the eye area in the gaze cue condition than in the no-gaze cue condition in the 0–0.2 s and 0.2–0.4 s time-windows (Fig. 2). The ANOVAs also revealed a significant effect of period (i.e., trial experience) in the 0–0.2 s and 0.2–0.4 s time-windows after onset. Specifically, the proportion of fixation times in the second 5 trials were significantly larger than those in the first 5 trials in the 0–0.2 s and 0.2–0.4 s time windows. Furthermore, the time to first fixation to the eye area, which has a significant negative correlation to the total gaze duration (during face image presentation), confirmed that the ASD group performed quicker, and had comparable responses to their TD peers, to the eye area in the second half of trials in this short task (while the ASD group had a significantly longer time to first fixation than their TD peers in the first half of trials). These results suggest that trial experience enhanced fixation behavior in the eye area in the early phase of the face presentation in both TD and ASD groups. The current results show that the participants with ASD effectively used gaze cues for the face-following task, which supports prior research^23,37^. We also found that experience with a relatively small number of trials (10 trials including 16 sequences, less than 15 min) facilitated this gaze cue use in individuals with ASD. In sum, valid use of gaze cues according to trial experience enhanced the eye fixation of participants with ASD in the early phases; however, the whole fixation time during presentation in the ASD group did not increase and was still around half of that displayed by their TD peers.

In the later phase of face presentation, the proportion of fixation times in the second 5 trials were significantly smaller than that in the first 5 trials during the 0.8–1.0 s time window, which indicates that both TD and ASD groups clearly showed a shorter fixation time in the second period than in the first period, which could result from an enhancement in anticipatory gaze behavior to the next face image through trial experience. Concerning anticipatory eye movements, Falck-Ytter^38^ showed that, in an action observation task in which participants were required to watch a video clip showing an actor’s reach-to-grasp and placement actions, young children with ASD (mean age: 5.1 years ± 10.5 months) could use predictive eye movements (attributed by the preceding gaze behavior to the actor’s action) as effectively as TD children and adults. However, von Hofsten, Uhlig, Adell, and Kochukhova^39^ found that children with ASD (aged 2:10 to 6:1 years) did not predict turn-taking in a videotaped conversation between two people.

Considering the diverse results of gaze behavior in individuals with ASD (from infants to adults), Guillon, Hadjikhani, Baduel, and Roge^40^ noted that social orienting is not qualitatively impaired; rather, it appears to be less efficient in ASD (see also Johnson^41^), and Guillon and colleagues have emphasized that the results might vary according to the task context (e.g. static vs. dynamic situations, and complexity). In the context of hand movements, Schmitz, Martineau, Barthelemy, and Assaiante^42^ revealed flexor inhibition when participants’ voluntary unloading started late after the onset of unloading in ASD children (aged 5.9 to 10.6 years); this inhibition started just before the onset of unloading in their TD peers, which indicates that there is an impairment of anticipatory postural adjustment in ASD when performing a voluntary bimanual load-lifting task. Fukui et al.^43^ also showed that an ASD group (mean age = 18.3 ± 2.1) experienced a significantly longer transition period from grasping end (i.e., stable holding when touching the surface of the object) to uplift initiation than did the TD group when performing unimanual reach-to-grasp and uplift movements, which suggests that those with ASD have difficulties chaining motor acts (see also Cattaneo et al.^44^; Fabbri-Destro, Cattaneo, Boria, and Rizzolatti^45^). In sum, although some studies have reported that people with ASD show impaired prediction function (see also Sinha et al.^46^) and/or difficulties in chaining motor actions, we found that people with ASD showed similar anticipatory gaze behavior to their TD peers in the face-following task. As described previously, the time to first fixation to the eye area of the presented face image, which showed quicker responses using the gaze cue in the second half of trials, also showed improved anticipatory gaze performance in the ASD group after a short-period experience.

Concerning the relationship between the face-following performance (according to the gaze cue and trial experience) and the total AQ score, the Index of gaze behavior (IGB) was calculated. The IGB indicates the effect of gaze cue modulated by trial experience on the fixation time in the eye area. The correlation analysis revealed a significant positive correlation in the 0–0.2 s time-window only, which suggests that the contribution of gaze cues (according to trial experience) to fixating on the eye area is greater for individuals with higher AQ scores in the early phase of face image presentation, regardless of whether an individual is TD or has ASD (Supplementary Fig. S2). This implies that those who show more autistic traits may benefit from short-duration (i.e., less than 15 min) gaze-cue training. On the other hand, it is difficult to affirm that the raw time difference (i.e., around 0.01 s) shown by the numerator of this index has significant meaning for improving daily communication skills. Therefore, future studies that combine other training applications^47^ should examine whether this task can improve social communication skills among those with ASD, and accumulate further knowledge about gaze behavior in those with ASD. In addition to the small sample size, one limitation of this study was use of a line-drawn face image, rather than a photograph of a face; this stimuli type was chosen because ASD participants reported that real faces (i.e., photograph of faces) in which the gaze was directed at them induced feelings of fear (see “Apparatus” section). When considering the real-world relevance, the next challenge is to manage ASD participants’ fear when performing this gaze-following task using real photographs, and to evaluate the effect of this “real version” task. However, as a first step towards a potential intervention, the use of a line-drawn face image that does not induce fear in ASD participants would be beneficial.

## Conclusions

Older adolescents and young adults with ASD looked less at the eye area in face images than did their TD peers; however, they could appropriately use gaze cues and their fixation on the eye area was increased after experience with a small number of trials. Furthermore, the total AQ score was associated with enhanced gaze-cue usage relating to trial experience, regardless of whether an individual was TD or had ASD.

## Methods

### Participants

Ten individuals (one woman) with ASD (including children of the members of an autistic community in Tokorozawa—“Yotsuba club”) and 10 TD peers (1 woman) participated in this experiment (see Table 1 for demographics). All participants except one with ASD were right handed, as assessed by the Edinburgh Handedness Inventory^48^, and had normal or corrected-to-normal vision. Participants were naive as to the purpose of the experiment and were paid for their participation. Owing to minimal verbal demands of the current task, groups were matched on non-verbal IQ, in addition to age, sex, and handedness. IQ assessments were conducted using a Japanese version^49^ of the Wechsler Adult Intelligence Scale-III (WAIS-III)^50^ and all participants’ non-verbal IQ scores exceeded 80.

**Table 1:** Demographic characteristics of participants with ASD and typically developing peers.

	Sex (M: F)		Age	IQ			AQ	ADOS-2 (Module 4)		
				Full	Non-verbal	Verbal		Comm	SI	Comm. + SI
ASD	9: 1	Range	16–23	85–122	91–105	76–134	13–42	2–6	4–10	6–14
		Mean	18.7	100.7	98.3	102.5	26.3	3.1	6.3	9.4
		SD	(2.1)	(11.8)	(4.7)	(16.8)	(9.1)	(1.3)	(2.3)	(2.7)
TD	9: 1	Range	16–24	82–131	82–123	85–135	12–24			
		Mean	19.8	112.9	103.3	118.1	17.3			
		SD	(2.7)	(13.0)	(11.9)	(15.0)	(4.5)			
			*t*(18) = − 1.006	*t*(18) = − 2.203	*t*(11.793) = − 1.236	*t*(18) = − 2.186	*t*(13.247) = 2.802			
			*p* = 0.328	*p = 0.041	*p* = 0.240	*p = 0.042	*p = 0.015			

*p < 0.05 (independent-samples t-test for comparison between ASD and TD groups).

Comm.: Communication score (cutoffs: 3/2), SI: Social Interaction score (cutoffs: 6/4), Comm. + SI: the summed score (Communication and Social Interaction) (cutoffs: 10/7). The cutoffs in parentheses denote the minimum scores for diagnosing autism and autism spectrum disorder, respectively.

A Japanese version^51^ of the Autism-Spectrum Quotient (AQ) test^52^ confirmed that no participants in the TD group had clinically significant levels of autistic traits (i.e., the AQ score was less than the cutoff of 33). Participants with ASD were diagnosed as having pervasive developmental disorder or ASD according to the Diagnostic and Statistical Manual of Mental Disorders, fourth edition^3^ or fifth edition^53^ by child psychiatrists. Their diagnoses were also verified by a Japanese version^54^ of the Autism Diagnostic Observation Schedule Second Edition Module 4 (ADOS-2)^55^. Although one participant in the ASD group was classified as non-spectrum according to the ADOS-2 criteria, this participant received a diagnosis from a child psychiatrist; therefore, this participant was included in the ASD group (furthermore, the participant’s exclusion did not alter the pattern of significance). No participants, except two with ASD, took any medications; one participant took 3 mg of aripiprazole per day, and the other took 100 mg of atomoxetine per day. Specific data on socioeconomic status and educational attainment levels were not collected. As mentioned above, some children from the “Yotsuba club” participated in this experiment; however, the members of the “Yotsuba club” were not involved in the study itself.

The study was approved by the institutional ethics committee at the National Rehabilitation Center for Persons with Disabilities, and all participants (and their parents if participants were aged < 20 years) provided written informed consent according to institutional guidelines conforming to the Declaration of Helsinki. All experiments were performed in accordance with relevant regulations and guidelines of the Ministry of Health, Labour and Welfare of Japan.

### Apparatus

As shown in Fig. 1A, a laptop PC (Thinkpad T440p, Lenovo Corporation, Beijing, China; 14 inch, screen resolution = 1280 × 720 pixels) was used for the presentation of face stimuli and data acquisition. Eye movements were recorded using a portable Tobii X2-30 compact eye-tracker with sampling at 30 Hz (Tobii AB, Danderyd, Sweden). This eye-tracker is considerably robust to head motion; therefore, a chin rest was not used to stabilize participants’ eye positions. To determine what face stimuli to utilize, we conducted informal interviews with a few individuals with ASD. They reported that real faces (i.e., photographs of faces) in which the gaze was directed at them could induce feelings of fear, and that they preferred line-drawn face images. Therefore, we employed line-drawn smiling face images (257 × 221 pixels; Fig. 1B) in the present experiment.

### Procedure

Participants were seated comfortably in a chair in front of a laptop PC, and the distance between their eyes and the screen was approximately 60 cm (Fig. 1A). The following task was implemented by a custom-made program (Solidray, Yokohama, Japan). Participants were required to track moving face images, which were sequentially presented for one second. Participants were not explicitly instructed to look at any specific part of the face image. As shown in Fig. 1C, there were 15 potential locations for the presented face images (i.e., 3 × 5), and the location at the beginning and end of each trial was always the center. Movements between locations were always horizontal or vertical, and were never diagonal. As shown in Fig. 1D, two types of face images were used, as follows: 1) the gaze of the face image was directed to participants (no-gaze cue condition) and 2) the gaze of the face image was directed to the position where the next face would be presented (gaze cue condition). The stimulus-onset asynchrony was set at 1 s and the inter stimulus interval was set at 0 ms. One trial consisted of 16 displacements, which included four distances of both directions (i.e., from left to right and from right to left) for horizontal displacements, and two distances of both directions (i.e., down and up) for vertical displacements (twice in each distance and direction in a trial) (Fig. 1C). Under these constraints, the “permutation” of the 16 sequences in each trial was different across all 10 trials.

The experimental session in each condition consisted of 10 trials. The no-gaze cue condition was the control condition. All participants first performed the no-gaze cue condition trials, and then completed the gaze cue condition trials to avoid contaminating performance in the control condition through experience in the gaze cue condition. The task took less than 15 min to complete.

### Data processing and analyses

Before calculating the proportion of fixation time to the eye area to valid gaze data in each 0.2 s time window during face image presentation, the two following analyses were performed. First, the duration in which fixation could not be detected by the eye-tracker was calculated, and the mean ratio of this duration to the one-second presentation time was computed. These values were entered into a two-way ANOVA with group (ASD, TD) as a between-participants factor and gaze-cue condition (gaze and no-gaze cue conditions) as a within-participant factor. Second, the number of sequences in which the fixation point never dropped within the eye area (i.e., the fixation time in the eye area was zero) was counted and the ratio of “zero-fixation to the eye area” sequences to the total number of sequences was computed separately for the first and second half of all 10 trials (i.e., 5 trials (80 sequences) each). To analyze this ratio, we performed a three-way ANOVA with group (ASD, TD) as a between-participants factor, and gaze-cue condition (gaze and no-gaze cue conditions) and period (first 5 trials and second 5 trials) as within-participant factors. To analyze the proportion of fixation time to the eye area, the data extracted from these two above-mentioned analyses were not included and other criteria for outlier deletion were not adopted.

The eye area in the face image was defined (red rectangle in Fig. 1B), and excluded the nose area (green rectangle in Fig. 1B). The fixation time in the eye area aligned by the presentation onset during the one-second presentation of each face stimulus was calculated for every 0.2 s (i.e., the mean fixation time in each 0.2 s time window) based on a previous eye-tracking study that investigated gaze behavior of individuals with and without ASD^56^. To verify the effects of group and gaze cue, and to investigate the effect of short-term experience, the mean proportion of fixation time in each 0.2 s time bin was entered into a three-way ANOVA with group (ASD, TD) as a between-participants factor, and the gaze-cue condition (gaze and no-gaze cue conditions) and period (first 5 trials and second 5 trials) as within-participant factors. The proportion per 0.2 s was calculated as follows:

Proportion of fixation time to eye area = *Fixation time to the eye area (s)/Valid fixation time on screen (s)*

Bonferroni-corrected post-hoc comparisons were performed when necessary. We performed an ANOVA for all time bins (i.e., 0–0.2, 0.2–0.4, 0.4–0.6, 0.6–0.8, and 0.8–1.0 s after stimuli presentation), and α = 0.05/5 = 0.01 was considered to be statistically significant, using the Bonferroni correction.

In addition to the mean proportion of fixation time to the eye area in each 0.2 s bin, we also analyzed the time to the first fixation to the eye area of the presented face images. A three-way ANOVA with group (ASD, TD) as a between-participants factor, and gaze-cue condition (gaze and no-gaze cue conditions) and period (first 5 trials and second 5 trials) as within-participant factors was conducted. To verify whether the total gaze duration to the eye area (during face image presentation) was associated with the time to the first fixation, and whether this correlation was different between the two groups, Fisher-transformed correlations (*r’*) between the total gaze duration and the time to first fixation for each condition in each participant were calculated. The *r’* for each condition in each participant was entered into a three-way ANOVA with group (ASD, TD) as a between-participants factor and the gaze-cue condition (gaze and no-gaze cue conditions) and period (first 5 trials and second 5 trials) as within-participant factors.

Furthermore, to determine whether individual autistic traits (i.e., total AQ score) were related to gaze behavior, we calculated Pearson’s correlation coefficients between the total AQ score and the index of gaze behavior (IGB, see below) in each time window by pooling data of both the ASD and TD groups. When computing each correlation coefficient, five levels (i.e., each 0.2 s time window) were set in the experimental design, such that α = 0.05/5 = 0.01 was considered to be statistically significant, using the Bonferroni correction. Our interest was whether the gaze cue contributes to fixation to the eye area, and whether any such contribution is enhanced by trial experience. Therefore, the IGB at each time window was calculated as follows. The numerator is assumed to compute the extent of fixation enhancement from the first half to the second period of trials, and the denominator gives a base condition for “normalized” comparisons between participants.

IGB = [*(mean fixation time of the second-half period in the gaze cue condition* *−* *mean fixation time of the first-half period in the gaze cue condition)* *−* *(mean fixation time of the second-half period in the no-gaze cue condition* *−* *mean fixation time of the first-half period in the no-gaze cue condition)*]/*(mean fixation time of the first-half period in the gaze cue condition *+ *mean fixation time of the first-half period in the no-gaze cue condition)*.

## Supplementary Information

Supplementary Information.

## References

[1] Kanner L (1943). Autistic disturbances of affective contact. Nerv. Child.

[2] Asperger, H. *'Autistic Pcychopathy' in Children (1944). Translated in U. Frith. Autism and Asperger's Syndrome* (Cambridge University Press, 1991).

[3] American Psychiatric Association. *Diagnostic and Statistical Manual of Mental Disorders *4th edn.,Text Revision. (American Psychiatric Association, 2000).

[4] Wing L (1981). Asperger's syndrome: a clinical account. Psychol. Med..

[5] Wing L, Gould J (1979). Severe impairments of social interaction and associated abnormalities in children: epidemiology and classification. J. Autism Dev. Disord..

[6] Baron-Cohen S, Belmonte MK (2005). Autism: a window onto the development of the social and the analytic brain. Annu. Rev. Neurosci..

[7] Frith U, Morton J, Leslie AM (1991). The cognitive basis of a biological disorder: autism. Trends Neurosci..

[8] Happé F, Frith U (2006). The weak coherence account: detail-focused cognitive style in autism spectrum disorders. J. Autism Dev. Disord..

[9] Senju A, Johnson MH (2009). Atypical eye contact in autism: models, mechanisms and development. Neurosci. Biobehav. Rev..

[10] Klin A, Jones W, Schultz R, Volkmar F, Cohen D (2002). Defining and quantifying the social phenotype in autism. Am. J. Psychiatry.

[11] Nakano T (2010). Atypical gaze patterns in children and adults with autism spectrum disorders dissociated from developmental changes in gaze behaviour. Proc. R. Soc. Lond. B. Biol. Sci..

[12] Pelphrey KA (2002). Visual scanning of faces in autism. J. Autism Dev. Disord..

[13] Phillips W, Baron-Cohen S, Rutter M (1992). The role of eye contact in goal detection: evidence from normal infants and children with autism or mental handicap. Dev. Psychopathol..

[14] Riby DM, Hancock PJB (2008). Viewing it differently: Social scene perception in Williams syndrome and Autism. Neuropsychologia.

[15] Dawson G (2004). Early social attention impairments in autism: social orienting, joint attention, and attention to distress. Dev. Psychol..

[16] Mundy P, Sigman M, Kasari C (1994). Joint attention, developmental level, and symptom presentation in autism. Dev. Psychopathol..

[17] Dalton KM (2005). Gaze fixation and the neural circuitry of face processing in autism. Nat. Neurosci..

[18] Mertens I, Siegmund H, Grusser OJ (1993). Gaze motor asymmetries in the perception of faces during a memory task. Neuropsychologia.

[19] Kylliäinen A, Hietanen JK (2006). Skin conductance responses to another person's gaze in children with autism. J. Autism Dev. Disord..

[20] Joseph RM, Ehrman K, McNally R, Keehn B (2008). Affective response to eye contact and face recognition ability in children with ASD. J. Int. Neuropsychol. Soc..

[21] Butterworth G, Jarrett N (1991). What minds have in common is space—spatial mechanisms serving joint visual-attention in infancy. Br. J. Dev. Psychol..

[22] Mundy P, Newell L (2007). Attention, joint attention, and social cognition. Curr. Dir. Psychol. Sci..

[23] Senju A, Tojo Y, Dairoku H, Hasegawa T (2004). Reflexive orienting in response to eye gaze and an arrow in children with and without autism. J. Child. Psychol. Psychiatry.

[24] Driver J (1999). Gaze perception triggers reflexive visuospatial orienting. Vis. Cogn..

[25] Fletcher-Watson S, Leekam SR, Benson V, Frank MC, Findlay JM (2009). Eye-movements reveal attention to social information in autism spectrum disorder. Neuropsychologia.

[26] Freeth M, Ropar D, Chapman P, Mitchell P (2010). The eye gaze direction of an observed person can bias perception, memory, and attention in adolescents with and without autism spectrum disorder. J. Exp. Child Psychol..

[27] Nation K, Penny S (2008). Sensitivity to eye gaze in autism: is it normal? Is it automatic? Is it social?. Dev. Psychopathol..

[28] Itier RJ, Batty M (2009). Neural bases of eye and gaze processing: the core of social cognition. Neurosci. Biobehav. Rev..

[29] Haswell CC, Izawa J, Dowell LR, Mostofsky SH, Shadmehr R (2009). Representation of internal models of action in the autistic brain. Nat. Neurosci..

[30] Leonard HC, Hill EL (2014). Review: The impact of motor development on typical and atypical social cognition and language: a systematic review. Child. Adolesc. Ment. Health.

[31] Casartelli L, Molteni M, Ronconi L (2016). So close yet so far: Motor anomalies impacting on social functioning in autism spectrum disorder. Neurosci. Biobehav. Rev..

[32] Jeannerod M (2006). Motor Cognition: What Actions Tell the Self.

[33] Bayliss AP, Tipper SP (2005). Gaze and arrow cueing of attention reveals individual differences along the autism spectrum as a function of target context. Br. J. Psychol..

[34] Hietanen JK (1999). Does your gaze direction and head orientation shift my visual attention?. NeuroReport.

[35] Cousineau D (2005). Confidence intervals in within-subject designs: a simpler solution to Loftus and Masson's method. Tutor Quant. Methods Psychol..

[36] Morey RD (2008). Confidence intervals from normalized data: A correction to Cousineau (2005). Tutor Quant. Methods Psychol..

[37] Vlamings PHJM, Stauder JEA, van Son IAM, Mottron L (2005). Atypical visual orienting to gaze- and arrow-cues in adults with high functioning autism. J. Autism Dev. Disord..

[38] Falck-Ytter T (2010). Young children with autism spectrum disorder use predictive eye movements in action observation. Biol. Lett..

[39] von Hofsten C, Uhlig H, Adell M, Kochukhova O (2009). How children with autism look at events. Res. Autism Spectr. Disord..

[40] Guillon Q, Hadjikhani N, Baduel S, Roge B (2014). Visual social attention in autism spectrum disorder: insights from eye tracking studies. Neurosci. Biobehav. Rev..

[41] Johnson MH (2014). Autism: demise of the innate social orienting hypothesis. Curr. Biol..

[42] Schmitz C, Martineau J, Barthelemy C, Assaiante C (2003). Motor control and children with autism: deficit of anticipatory function?. Neurosci. Lett..

[43] Fukui T (2018). Older adolescents and young adults with autism spectrum disorder have difficulty chaining motor acts when performing prehension movements compared to typically developing peers. Front. Hum. Neurosci..

[44] Cattaneo L (2007). Impairment of actions chains in autism and its possible role in intention understanding. Proc. Natl. Acad. Sci. USA.

[45] Fabbri-Destro M, Cattaneo L, Boria S, Rizzolatti G (2009). Planning actions in autism. Exp. Brain Res..

[46] Sinha P (2014). Autism as a disorder of prediction. Proc. Natl. Acad. Sci. USA.

[47] Wieckowski AT, White SW (2017). Application of technology to social communication impairment in childhood and adolescence. Neurosci. Biobehav. Rev..

[48] Oldfield RC (1971). The assessment and analysis of handedness: the Edinburgh inventory. Neuropsychologia.

[49] Fujita K, Maekawa H, Dairoku K, Yamanaka K (2006). A Japanese Version of the WAIS-III.

[50] Wechsler D (1997). Wechsler Adult Intelligence Scale-III.

[51] Wakabayashi A, Tojo Y, Baron-Cohen S, Wheelwright S (2004). The Autism-Spectrum Quotient (AQ) Japanese version: evidence from high-functioning clinical group and normal adults. Shinrigaku Kenkyu.

[52] Baron-Cohen S, Wheelwright S, Skinner R, Martin J, Clubley E (2001). The autism-spectrum quotient (AQ): evidence from Asperger syndrome/high-functioning autism, males and females, scientists and mathematicians. J. Autism Dev. Disord..

[53] American Psychiatric Association (2013). Diagnostic and statistical manual of mental disorders.

[54] Kuroda M, Inada N (2015). Autism Diagnostic Observation Schedule, Second Edition, Japanese Version.

[55] Lord C (2012). Autism Diagnostic Observation Schedule.

[56] Neumann D, Spezio ML, Piven J, Adolphs R (2006). Looking you in the mouth: abnormal gaze in autism resulting from impaired top-down modulation of visual attention. Soc. Cogn. Affect. Neurosci..

