# Supplementary material for: Enhanced use of gaze cue in a face-following task after brief trial experience in individuals with autism spectrum disorder
Source: Sci Rep. 2021 May 27;11:11240. doi: 10.1038/s41598-021-90230-6 (PMC8160015; doi:10.1038/s41598-021-90230-6)
Supplement: Supplementary file 1 — Supplementary Information. [file 41598_2021_90230_MOESM1_ESM.docx]

Supplementary Information for

**Enhanced use of gaze cue in a face-following task after brief trial experience in individuals with autism spectrum disorder**

Takao Fukui^1,†*^, Mrinmoy Chakrabarty^1,ǂ^, Misako Sano^1,2^, Ari Tanaka^1^, Mayuko Suzuki^3^, Sooyung Kim^3^, Hiromi Agarie^3^, Reiko Fukatsu^1,3^, Kengo Nishimaki^2,3^, Yasoichi Nakajima^1,§,‡^, Makoto Wada^1*^

^1^Department of Rehabilitation for Brain Functions, Research Institute, National Rehabilitation Center for Persons with Disabilities, Tokorozawa, Japan

^2^Information and Support Center for the Persons with Developmental Disabilities, National Rehabilitation Center for Persons with Disabilities, Tokorozawa, Japan

^3^Department of Medical Treatment III (Pediatric and Child Psychiatric Section), Hospital, National Rehabilitation Center for Persons with Disabilities, Tokorozawa, Japan

^†^(present address) Faculty of Systems Design, Tokyo Metropolitan University, Hino, Japan

^ǂ^(present address) Department of Social Sciences and Humanities, Indraprastha Institute of Information Technology Delhi, New Delhi, India

^§^(present address) National Rehabilitation Center for Persons with Disabilities, Tokorozawa, Japan

^‡^(present address) Community Health Care Research Center, Nagano University of Health and Medicine, Nagano, Japan

*Correspondence: Takao Fukui (E-mail: takao-fukui@tmu.ac.jp),

Makoto Wada (E-mail: wada-makoto@rehab.go.jp)


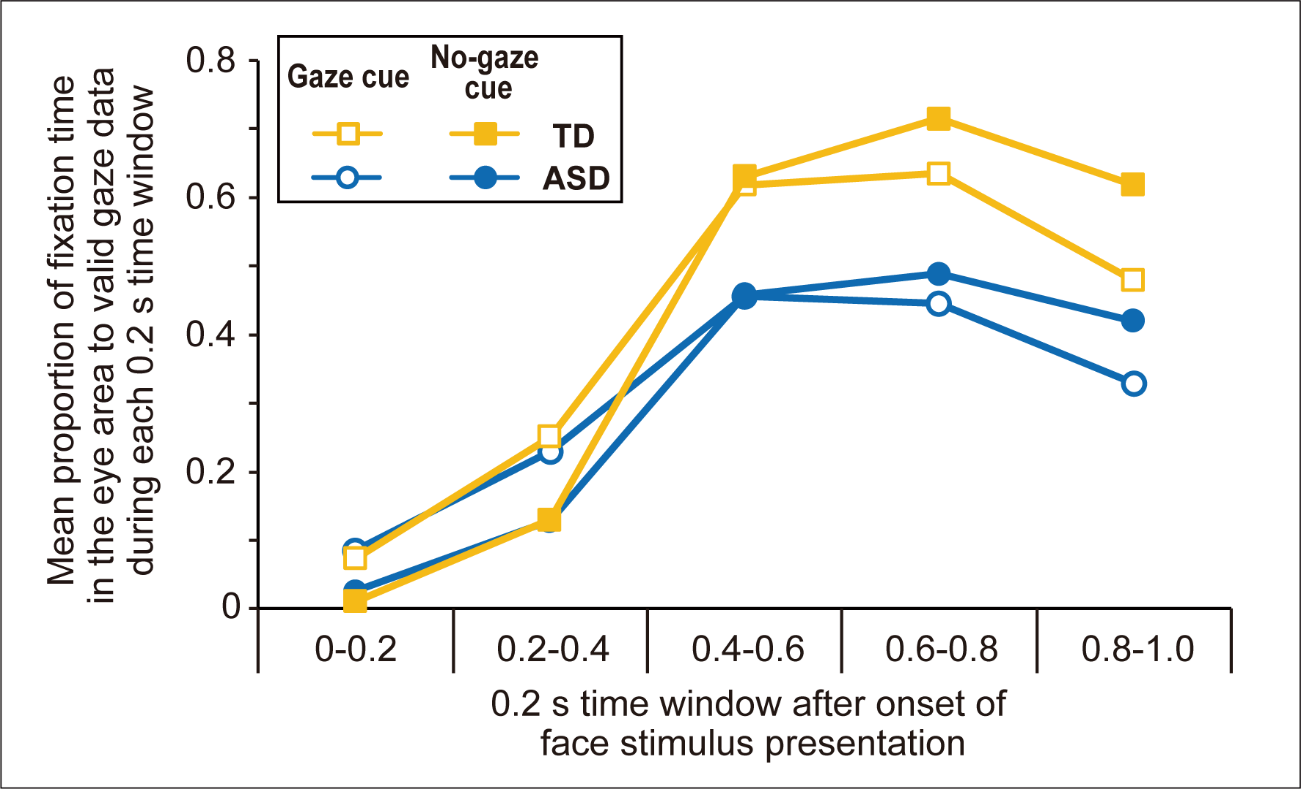


**Supplementary Figure S1. Mean proportion of fixation time in the eye area to valid gaze data during each 0.2 s time window calculated over all 10 trials (for legibility).** The proportion of fixation time in the eye area in the ASD group diverged from that in the TD group from around the second half of the one-second face image presentation.


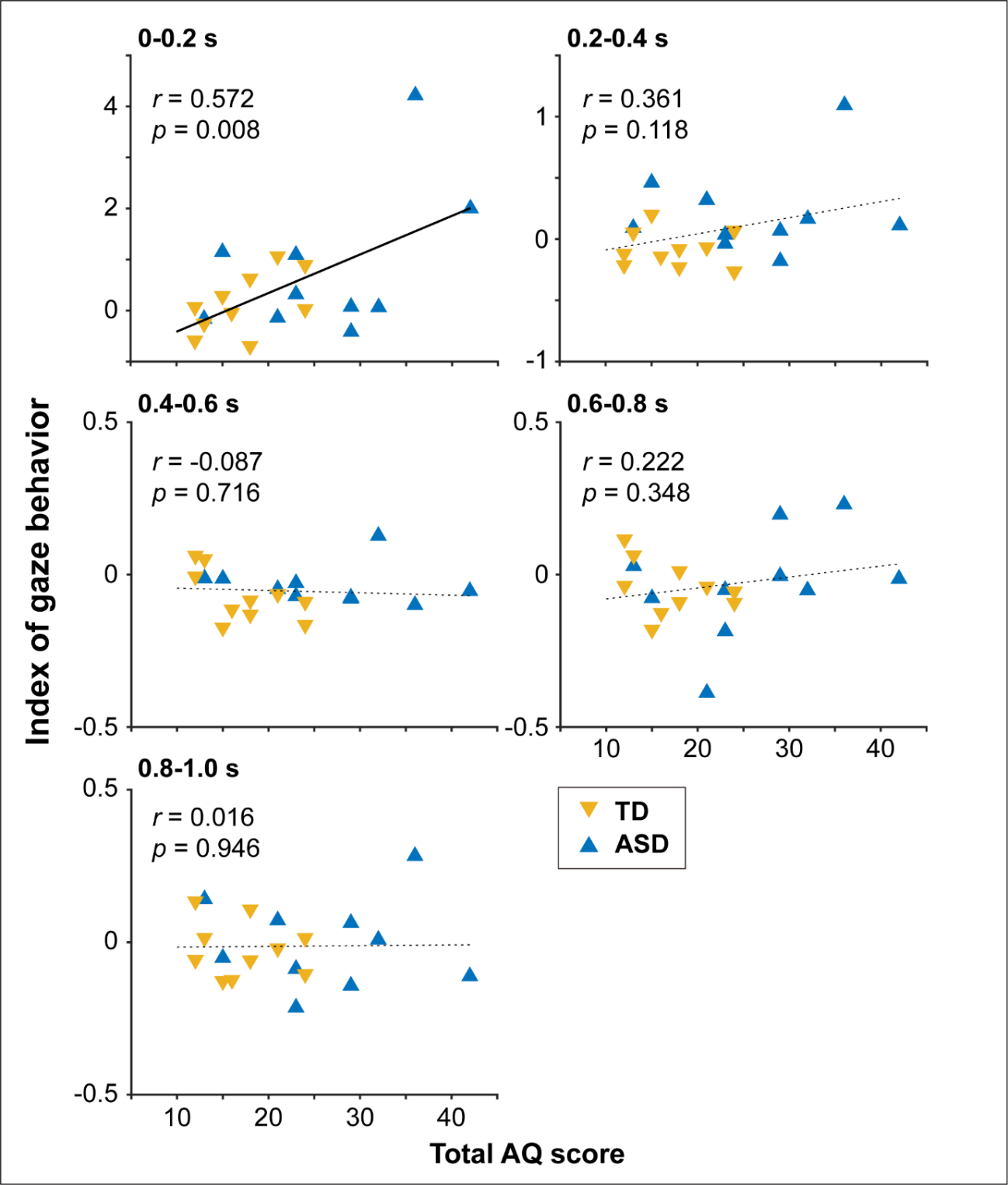


**Supplementary Figure S2. Correlation between index of gaze behavior and the total AQ score in each 0.2 s time window.** A significant positive relationship between enhanced appropriate gaze cue use and the total AQ score was found in the 0-0.2 s time window only, by pooling data of participants with ASD and typically developing individuals. The result suggests that appropriate gaze use in the early phase of each sequence was enhanced in individuals with more autistic traits, which resulted from the benefit of the short-period trial experiment.
